# X‐Box binding protein 1 downregulates SIRT6 to promote injury in pancreatic ductal epithelial cells

**DOI:** 10.1002/iid3.1301

**Published:** 2024-07-05

**Authors:** Zhuo Yang, Shaojun Li, Chuan Zhao, Zongzheng Zhao, Juan Tan, Lu Zhang, Yuanqing Huang

**Affiliations:** ^1^ Intensive Care Unit, Bazhong Hospital of Traditional Chinese Medicine Bazhong Sichuan China; ^2^ Acupuncture and Rehabilitation Department Bazhong Hospital of Traditional Chinese Medicine Bazhong Sichuan China

**Keywords:** ER stress, pancreatitis, pathology, sirtuin, transcription

## Abstract

**Objective:**

Acute pancreatitis (AP) stands as a frequent cause for clinical emergency hospital admissions. The X‐box binding protein 1 (XBP1) was found to be implicated in pancreatic acinar cell apoptosis. The objective is to unveil the potential mechanisms governed by XBP1 and SIRT6 in the context of AP.

**Methods:**

Caerulein‐treated human pancreatic duct epithelial (HPDE) cells to establish an in vitro research model. The levels and regulatory role of SIRT6 in the treated cells were evaluated, including its effects on inflammatory responses, oxidative stress, apoptosis, and endoplasmic reticulum stress. The relationship between XBP1 and SIRT6 was explored by luciferase and ChIP experiments. Furthermore, the effect of XBP1 overexpression on the regulatory function of SIRT6 on cells was evaluated.

**Results:**

Caerulein promoted the decrease of SIRT6 and the increase of XBP1 in HPDE cells. Overexpression of SIRT6 slowed down the secretion of inflammatory factors, oxidative stress, apoptosis level, and endoplasmic reticulum stress in HPDE cells. However, XBP1 negatively regulated SIRT6, and XBP1 overexpression partially reversed the regulation of SIRT6 on the above aspects.

**Conclusion:**

Our study illuminates the role of XBP1 in downregulating SIRT6 in HPDE cells, thereby promoting cellular injury. Inhibiting XBP1 or augmenting SIRT6 levels holds promise in preserving cell function and represents a potential therapeutic avenue in the management of AP.

## Graphical abstract

Based on JASPAR, XBP1 is thought to bind to the SIRT6 promoter for regulation. XBP1 levels were found to be significantly upregulated under caerulein treatment and negatively regulate SIRT6.

## 1 INTRODUCTION

Acute pancreatitis (AP), particularly acute necrotizing pancreatitis, represents a prevalent clinical acute inflammatory condition, characterized primarily by acute abdominal pain.^1^ It exhibits varying degrees of involvement in adjacent tissues and multiple organs, occasionally leading to systemic inflammatory response syndrome (SIRS) and multiple organ dysfunction (MODS).^2^ Consequently, it stands as a frequent cause for clinical emergency hospital admissions.^3^ There are currently no specific therapies that can reduce or prevent this condition, other than supportive care until inflammation subsides.^4^ In recent decades, considerable research efforts by scholars have significantly advanced our understanding of the multifaceted and intricate pathophysiological processes underlying AP. There is evidence of early‐onset immunosuppression in AP,^5^ and inflammatory monocytes and macrophages determine the severity of this disease.^6^ One widely acknowledged pathological mechanism is the premature activation of trypsinogen within acinar cells, resulting in extensive necrosis of pancreatic acinar cells.^7^, ^8^ While research on AP predominantly centers on pancreatic acinar cells, some studies encompass extra‐pancreatic tissues and organs. Nonetheless, there exists a paucity of research focusing on another critical functional component within pancreatic tissue, the pancreatic ductal mucosal barrier (PDMB).^9^ When provoked by inflammatory factors, sepsis, chemicals, and similar stimuli, the PDMB sustains disruption, causing edema and harm to pancreatic tissue, thus exacerbating AP.^10^

A comprehensive elucidation of the pathogenesis will greatly facilitate the development of effective treatment strategies. SIRT6, a nuclear protein belonging to the sirtuin family, possesses ADP‐ribosyltransferase and NAD + ‐dependent sirtuin activities.^11^ A growing body of research underscores its active role in protecting cells or tissues in inflammatory diseases. For instance, in a mouse model of acute lung injury, the overexpression of SIRT6 has been shown to reduce pathological lung injury and mitigate symptoms of pulmonary edema.^12^ Studies employing gene‐specific knockout and transgenic mouse models have revealed that hepatic SIRT6 deficiency exacerbates ethanol‐induced liver injury and hepatic endoplasmic reticulum (ER) stress.^13^ Furthermore, reduced levels of SIRT6 have been observed in the islets of diabetic and aged mice, with overexpression of SIRT6 proving beneficial in maintaining cell viability and augmenting glucose‐stimulated insulin secretion.^14^ However, the function of SIRT6 in AP remains enigmatic. Analysis on the JASPAR website exhibits that X‐box binding protein 1 (XBP1) binds to the SIRT6 promoter and has the potential function to regulate its transcriptional expression. The XBP1 signaling has been implicated in pancreatic acinar cell apoptosis,^15^ prompting the hypothesis that XBP1 participates in AP by modulating SIRT6.

The primary inducer for AP in experimental studies is cerulean.^16^ This investigation aims to employ caerulein to induce human pancreatic duct epithelial (HPDE) cells, establishing an in vitro research model. The objective is to unveil the potential mechanisms governed by XBP1 and SIRT6 in the context of AP. This endeavor lays the groundwork for understanding the pathological mechanism of AP in finer detail.

## 2 MATERIALS AND METHODS

### 2.1 Cell culture and treatment

HPDE cells (OriCell) were cultured in Dulbecco's modified Eagle's medium (DMEM; Gibco) with 10% fetal bovine serum, and 1% penicillin‐streptomycin mixture. The culture conditions were 37°C and 5% CO_2_. HPDE cells were stimulated with caerulein (10 nmol/L, sigma) for 12 h to mimic pathological state in vitro. Untreated cells served as a control group. For mechanistic studies, HPDE cells were transfected to promote SIRT6 overexpression or XBP1 knockdown. Briefly, pcDNA3.1 plasmids (SnapGene) carrying SIRT6 and XBP1‐targeted short hairpin RNAs (shRNAs; WZ Biosciences) were mixed separately with Lipofectamine™ 2000 transfection reagent (Invitrogen), and then the mixture was added to the cell wells. Cells transfected with empty plasmids or nontargeted shRNAs served as negative control groups. After 48 h of incubation, cells were harvested to verify transfection efficiency.

### 2.2 Reverse transcription‐quantitative polymerase chain reaction (RT‐qPCR)

TRIzol® Reagent (Invitrogen) was supplemented to the harvested HPDE cells, and total RNA was precipitated by a two‐step method of chloroform and isopropanol. RNA was converted to cDNA using an Evo M‐MLV reverse transcription kit (AG11707; Accurate), and the qPCR reactions were performed according to the instructions of the SYBR Green PCR kit (BL698A; Biosharp). Relative mRNA levels were measured using the ΔΔCt method after normalization to actin.

### 2.3 Western blot analysis

RIPA lysis buffer (Beyotime) was supplemented to the harvested HPDE cells, and proteins were isolated and quantified using a Nano 3000 detector (YPH‐Bio). Proteins were separated using SDS‐polyacrylamide gel electrophoresis and transferred to PVDF membranes (Roche). Next, membranes were blocked with 5% skimmed milk, followed by the incubation with primary antibodies against SIRT6, XBP1, ER stress‐related, apoptosis‐related proteins and HRP‐conjugated secondary antibody (Proteintech group or Abcam). Blots were visualized with ECL reagent (Biosharp) and gray values were analyzed using ImageJ software.

### 2.4 Enzyme‐linked immunosorbent assay (ELISA)

The inflammatory response was assessed by measuring TNF‐α, IL‐1β, and IL‐6 levels in cell supernatants. This assay was performed using ELISA kits (Beyotime) according to the manufacturer's instructions. Supernatant samples were centrifuged at 300*g* for 5 min. OD was recorded at 450 nm using a microplate reader. The corresponding concentration of the sample was calculated through the absorbance value of the sample and the standard curve.

### 2.5 2,7‐Dichlorodihydrofluorescein diacetate (DCFH‐DA) probe

The DCFH‐DA probe (Maokangbio) was used to quantify ROS levels in HPDE cells. DCFH‐DA is hydrolyzed by intracellular esterases to generate DCFH. ROS can oxidize DCFH to generate fluorescent DCF, which reflects the level of ROS. Caerulein‐treated cells were incubated with diluted working solution for 30 min and then washed with PBS. Cells were photographed under a fluorescence microscope (Nikon).

### 2.6 Oxidative stress

Oxidative stress was assessed by measuring malondialdehyde (MDA) level (S0131), superoxide dismutase (SOD, S0101) and catalase (CAT, S0051; Beyotime) activities. HPDE cells were lysed and centrifuged at 10,000*g* for 10 min to obtain the supernatant. The detections were performed according to the instructions of these commercial kits. The values were calculated according to the absorbance and the standard curve.

### 2.7 Flow cytometry

Flow cytometry was used to detect apoptosis with Annexin V‐FITC/PI apoptosis detection kit (Vazyme). Harvested HPDE cells were washed twice with cold PBS and suspended in binding buffer. The cell suspension was incubated with Annexin V FITC and PI for 15 min in the dark. Apoptosis was analyzed using flow cytometry (BD FACSCanto).

### 2.8 Caspase 3 activity

As mentioned above, the supernatant of the cell lysate was collected and the protein concentration was measured. Detection buffer, sample, and 10 μL of Ac‐DEVD‐pNA (2 mM; Beyotime) were sequentially added and mixed. A405 was measured after incubation at 37°C for 1–2 h.

### 2.9 Luciferase assay

Luciferase assay was used to determine SIRT6 promoter activity. The pGL3 luciferase reporter plasmid (Promega) carrying the SIRT6 promoter region (mutant and wild types) along with the XBP1 overexpression plasmid were separately transfected into HPDE cells using Lipofectamine™ 2000. Cells were harvested 24 h after transfection, and luciferase activity was measured by a dual‐luciferase reporter assay system (Promega).

### 2.10 Chromatin immunoprecipitation assay (ChIP)

Binding of XBP1 to the SIRT6 promoter was determined using ChIP. HPDE cells were fixed with 1% methanol and the cross‐linking reaction was terminated by 0.125 mol/L glycine. After washing, the cells were resuspended in SDS lysis solution, and the DNA was disrupted by ultrasonication. After centrifugation at 14,000 rpm for 10 min at 4°C, the sonicated chromatin liquid supernatant was collected. The supernatant was then incubated overnight with anti‐XBP1 or IgG antibody (Invitrogen). Protein A/G Agarose beads (Pierce) were added to harvest protein‐DNA complexes. An additional 50 μL of supernatant was taken without antibody addition as a negative control. DNA was retrieved by 5 mmol/L NaCl and purified by phenol/chloroform extraction, and the enrichment was determined using RT‐qPCR.

### 2.11 Immunofluorescence assay

HPDE cells were subjected to 4% formaldehyde for immobilization and 0.1% Triton X‐100 for permeabilization. Afterward, cells were blocked with 5% BSA for 30 min, and CHOP antibody (ab233121; Abcam) overnight at 4°C, followed by FITC‐labeled anti‐mouse antibody (ab6785; Abcam) for 1 h. After counterstaining with DAPI for 5 min for the nuclei, and images were captured under a fluorescence microscope (Nikon).

### 2.12 Statistical analysis

Data are presented as mean ± standard deviation (*n* ≥ 3) and statistical analysis was performed using SPSS 19.0. Statistical differences between groups were analyzed by unpaired *t*‐test (two groups) and one‐way ANOVA with Tukey's post hoc test (multiple groups). *p* < .05 indicates significant difference.

## 3 RESULTS

### 3.1 SIRT6 levels upon caerulein treatment

**Figure 1:**
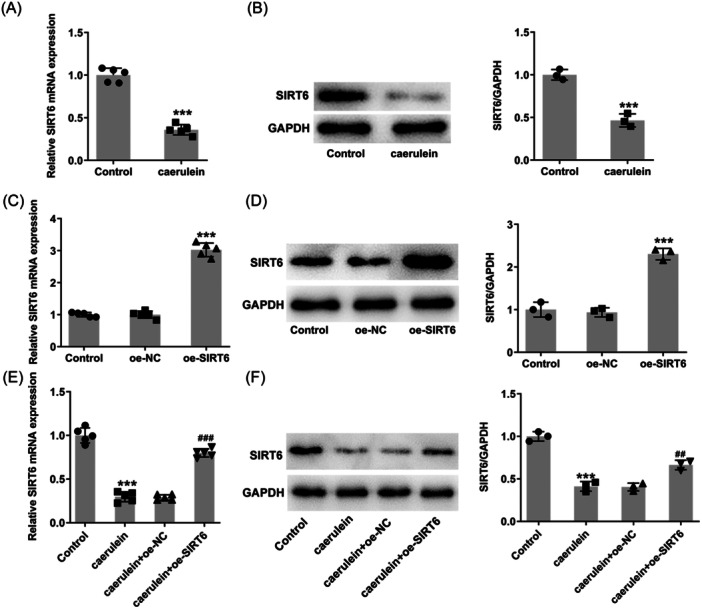
SIRT6 levels in HPDE cells upon caerulein treatment. (A) Reverse transcription‐quantitative polymerase chain reaction (RT‐qPCR) and (B) western blot analysis reveal SIRT6 level upon caerulein treatment. (C) RT‐qPCR and (D) western blot analysis reveal SIRT6 level after transfection. (E) RT‐qPCR and (F) western blot analysis reveal SIRT6 level upon caerulein treatment and transfection. ***p < .001 versus control; ^##^ *p* < .01, ^###^ *p* < .001 versus caerulein + oe‐NC.

SIRT6 levels were significantly decreased in caerulein‐treated cells compared to the control group, according to RT‐qPCR and western blot analysis results (Figure 1A,B). To study the regulatory role of SIRT6, overexpression of SIRT6 in cells was promoted by transfection. In the analysis of transfection results, SIRT6 levels were successfully increased in cells transfected with the specific overexpression plasmid (Figure 1C,D). Compared with the oe‐NC group, the level of SIRT6 in the oe‐SIRT6 group was increased after treatment with caerulein, while it was still lower than that in the control group (Figure 1E,F).

### 3.2 SIRT6 reduces inflammation and oxidative stress

**Figure 2:**
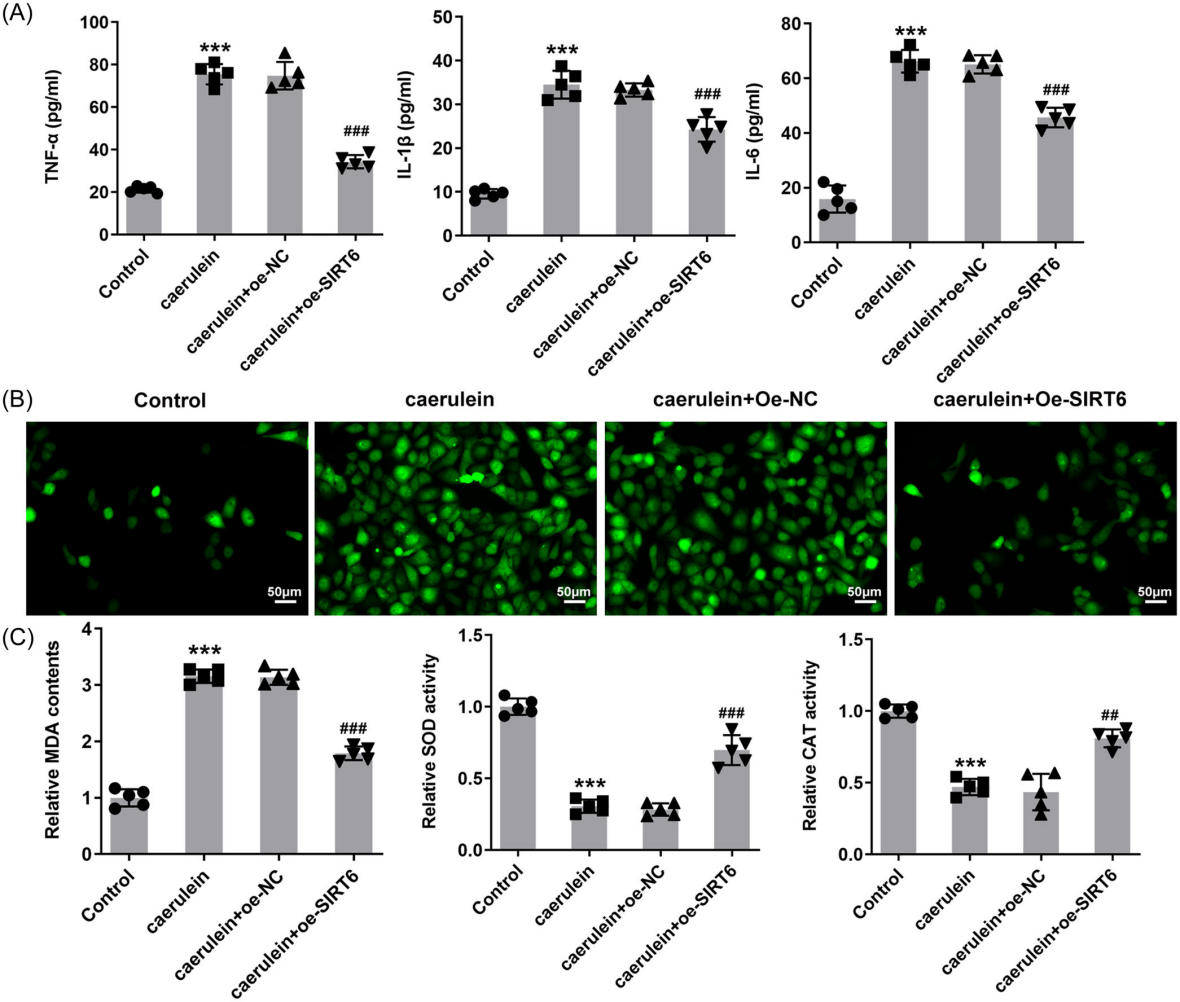
SIRT6 reduces the inflammation and oxidative stress in human pancreatic duct epithelial (HPDE) cells. (A) The levels of inflammatory factors TNF‐α, IL‐1β, and IL‐6 in the cell supernatant were measured. (B) The dichlorodihydrofluorescein diacetate (DCFH‐DA) probe was used to quantify reactive oxygen species (ROS) levels in HPDE cells, magnification 200×. (C) Oxidative stress was assessed by measuring malondialdehyde (MDA) level, superoxide dismutase (SOD) and catalase (CAT) activities. ***p < .001 versus control; ^##^ *p* < .01, ^###^ *p* < .001 versus caerulein + oe‐NC.

The levels of inflammatory factors TNF‐α, IL‐1β, and IL‐6 in the cell supernatant increased significantly in response to caerulein treatment. Compared with the caerulein + oe‐NC group, the levels of these inflammatory factors in the supernatant of cells overexpressing SIRT6 were reduced (Figure 2A). This trend was also reflected in ROS levels in cells. Compared with the control group, ROS levels increased significantly in the caerulein‐treated group, and additional SIRT6 overexpression effectively slowed down the increase in ROS (Figure 2B). The results also showed that caerulein induced the increase of MDA content and the decrease of SOD and CAT activities in the cells. The MDA in SIRT6 overexpressing cells treated with caerulein only increased slightly, and the SOD and CAT activities did not decrease severely (Figure 2C).

### 3.3 SIRT6 reduces the apoptosis

**Figure 3:**
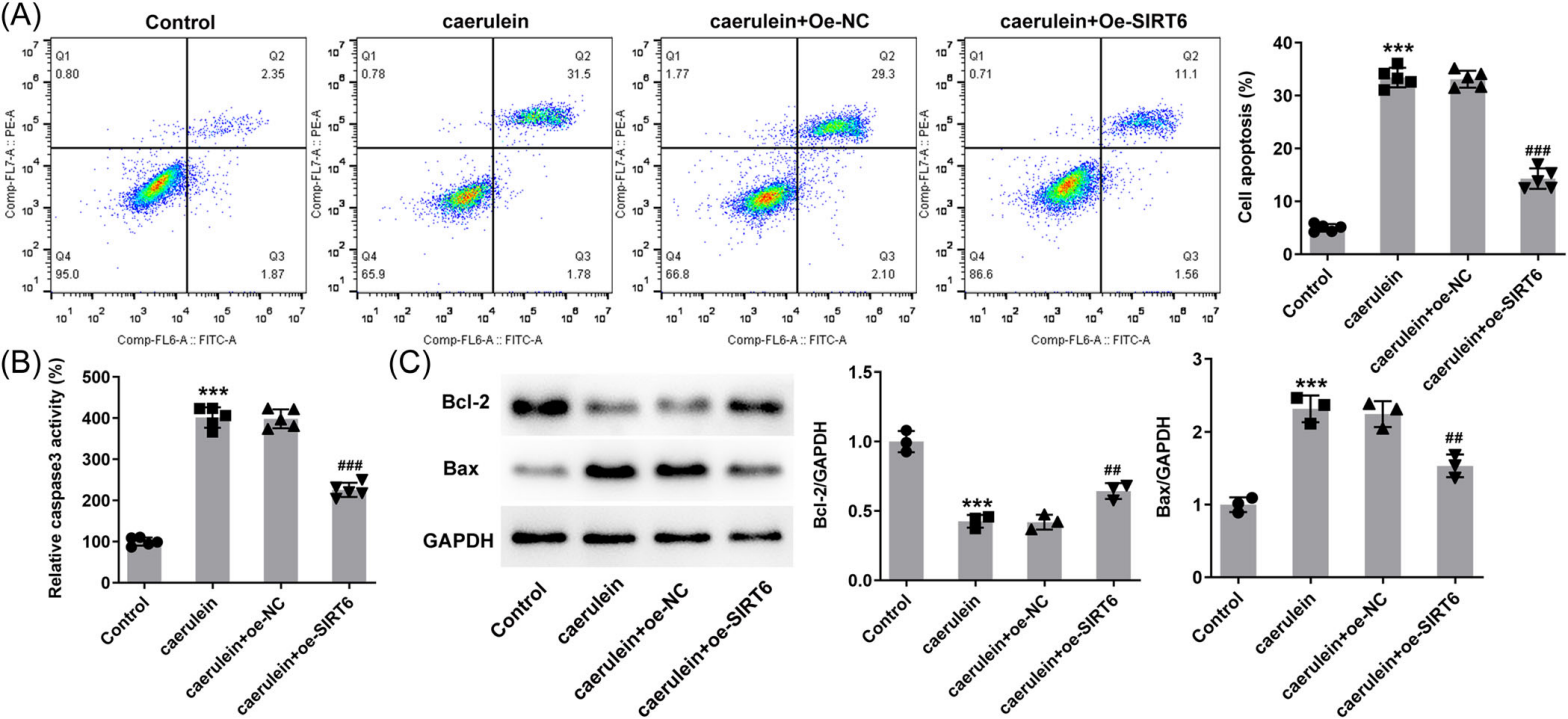
SIRT6 reduces the apoptosis in human pancreatic duct epithelial (HPDE) cells. (A) Flow cytometry was used to detect cell apoptosis. (B) Caspase 3 activity was also used to assess apoptosis. (C) Western blot analysis reveals the protein levels of Bcl‐2 and Bax. ***p < .001 versus control; ^##^ *p* < .01, ^###^ *p* < .001 versus caerulein + oe‐NC.

Flow cytometric analysis results revealed that caerulein treatment promoted cell apoptosis, and the apoptosis rate increased from ~ 4% to 33%. SIRT6 overexpression tended to protect cell survival and reduced the apoptosis rate to ~12% (Figure 3A). Specifically, caerulein increased the activity of caspase 3 in cells, and the increase in caspase 3 activity in cells overexpressing SIRT6 was significantly weakened (Figure 3B). In addition, western blot analysis results demonstrated that caerulein reduced intracellular Bcl‐2 protein levels and increased Bax protein levels, and additional SIRT6 overexpression reduced these effects (Figure 3C).

### 3.4 XBP1 negatively regulates SIRT6

**Figure 4:**
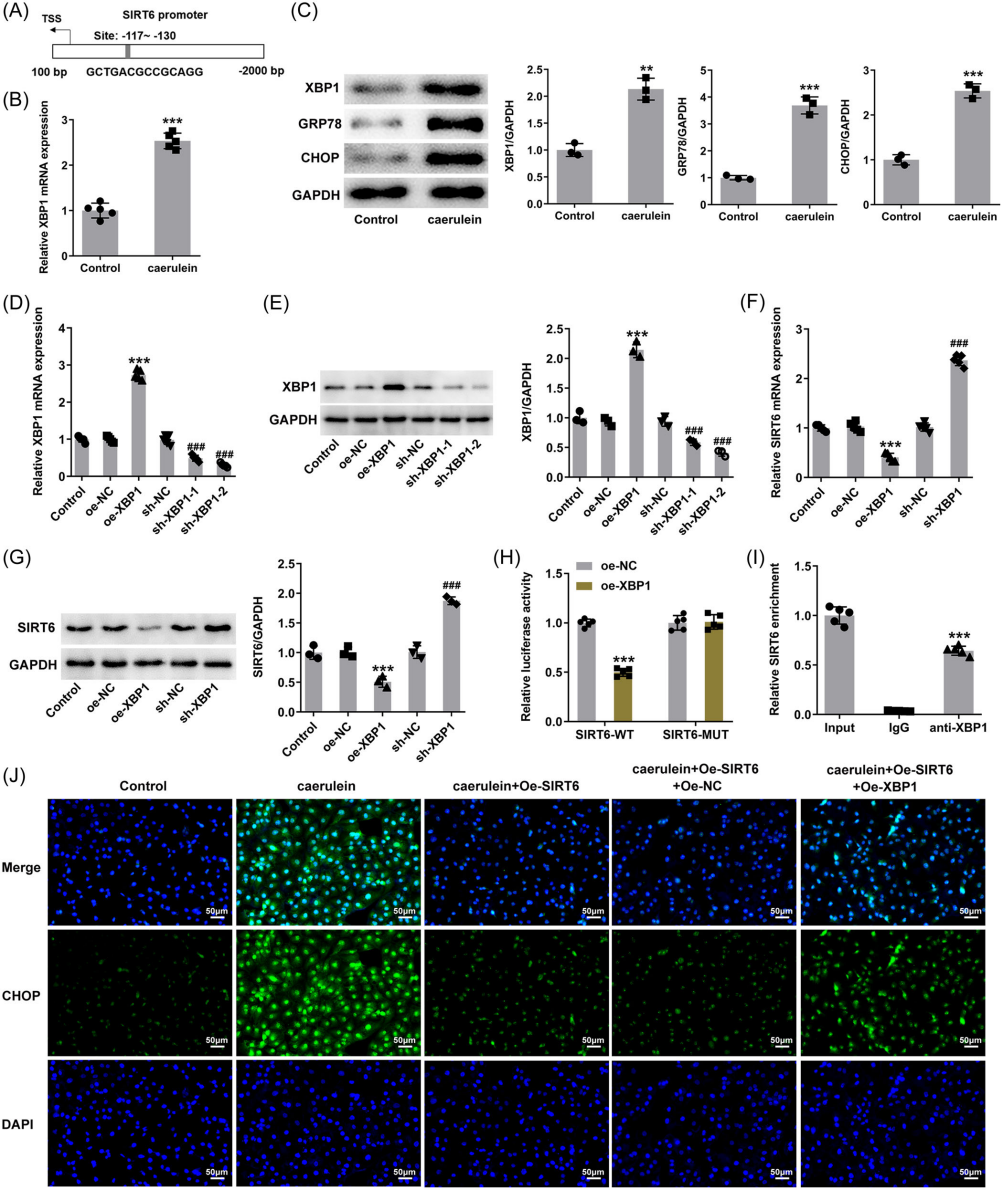
X‐Box binding protein 1 (XBP1) negatively regulates SIRT6. (A) Site of XBP1 binding to the SIRT6 promoter according to the JASPAR website. (B) Reverse transcription‐quantitative polymerase chain reaction (RT‐qPCR) reveals XBP1 level upon caerulein treatment. (C) Western blot analysis reveals XBP1, GRP78, and CHOP protein levels upon caerulein treatment. (D) RT‐qPCR and (E) western blot analysis reveal XBP1 level after transfection. (F) RT‐qPCR and (G) western blot analysis reveal SIRT6 level in response to XBP1 overexpression or knockdown. (H) Luciferase assay was used to determine SIRT6 promoter activity. (I) Binding of XBP1 to the SIRT6 promoter was determined using chromatin immunoprecipitation (ChIP). (J) The level of CHOP in human pancreatic duct epithelial (HPDE) cells was determined using immunofluorescence assay, magnification 200×. **p < .01, ***p < .001 versus control or oe‐NC or lgG; ^###^ *p* < .001 versus sh‐NC.

Based on JASPAR, XBP1 is thought to bind to the SIRT6 promoter for regulation, with the binding site indicating in Figure 4A, therefore the level of XBP1 in cells was determined. XBP1 levels were found to be significantly upregulated under caerulein treatment (Figure 4B). Meanwhile, intracellular GRP78 and CHOP protein levels also increased (Figure 4C). To reveal the regulatory mechanism of XBP1 in cells, cells were transfected to achieve XBP1 overexpression or knockdown (Figure 4D,E). Among them, the knockdown efficiency in cells transfected with sh‐XBP1‐2 was better and was used in subsequent experiments. Overexpression of XBP1 reduced the level of SIRT6, while the level of SIRT6 increased in the case of knockdown (Figure 4F,G), indicating that XBP1 and SIRT6 are in a negative regulatory relationship. In the luciferase assay, the SIRT6 promoter activity in the group transfected with the mutated promoter site reporter plasmid was no different from the control group. The difference was that the promoter activity of the group transfected with wild type decreased, indicating that XBP1 negatively regulated SIRT6 promoter activity (Figure 4H). The enrichment of SIRT6 in the anti‐XBP1 group in the ChIP experiment indicated that XBP1 could bind to the SIRT6 promoter (Figure 4I). Compared with simple transfection of SIRT6 overexpression plasmid and empty plasmid, simultaneous transfection with SIRT6 and XBP1 overexpression plasmids could cause an increase in CHOP protein level (Figure 4J).

**Figure 5:**
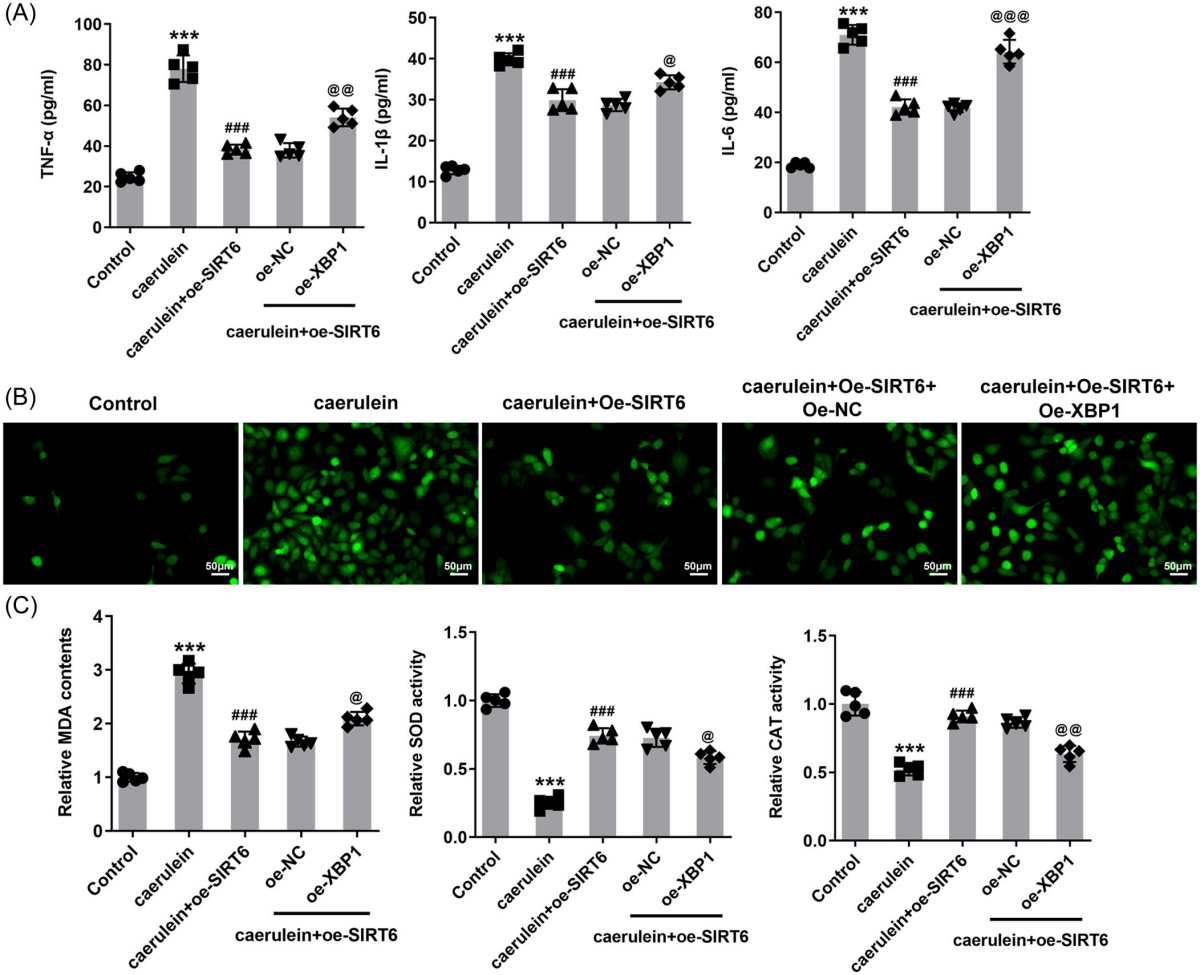
X‐Box binding protein 1 (XBP1) curbs the impacts of SIRT6 on the inflammation and oxidative stress. Cells were subjected to simultaneous transfection with SIRT6 and XBP1 overexpression plasmids. (A) the levels of inflammatory factors in the cell supernatant were measured upon caerulein treatment. (B) The dichlorodihydrofluorescein diacetate (DCFH‐DA) probe was used to quantify reactive oxygen species (ROS) levels in human pancreatic duct epithelial (HPDE) cells, magnification 200×. (C) Oxidative stress was assessed by measuring malondialdehyde (MDA) level, superoxide dismutase (SOD), and catalase (CAT) activities. ***p < .001 versus control; ^###^ *p* < .001 versus caerulein; ^@^ *p* < .05, p@@ < .01, ^@@@^ *p* < .001 versus caerulein + oe‐SIRT6 + oe‐NC.

**Figure 6:**
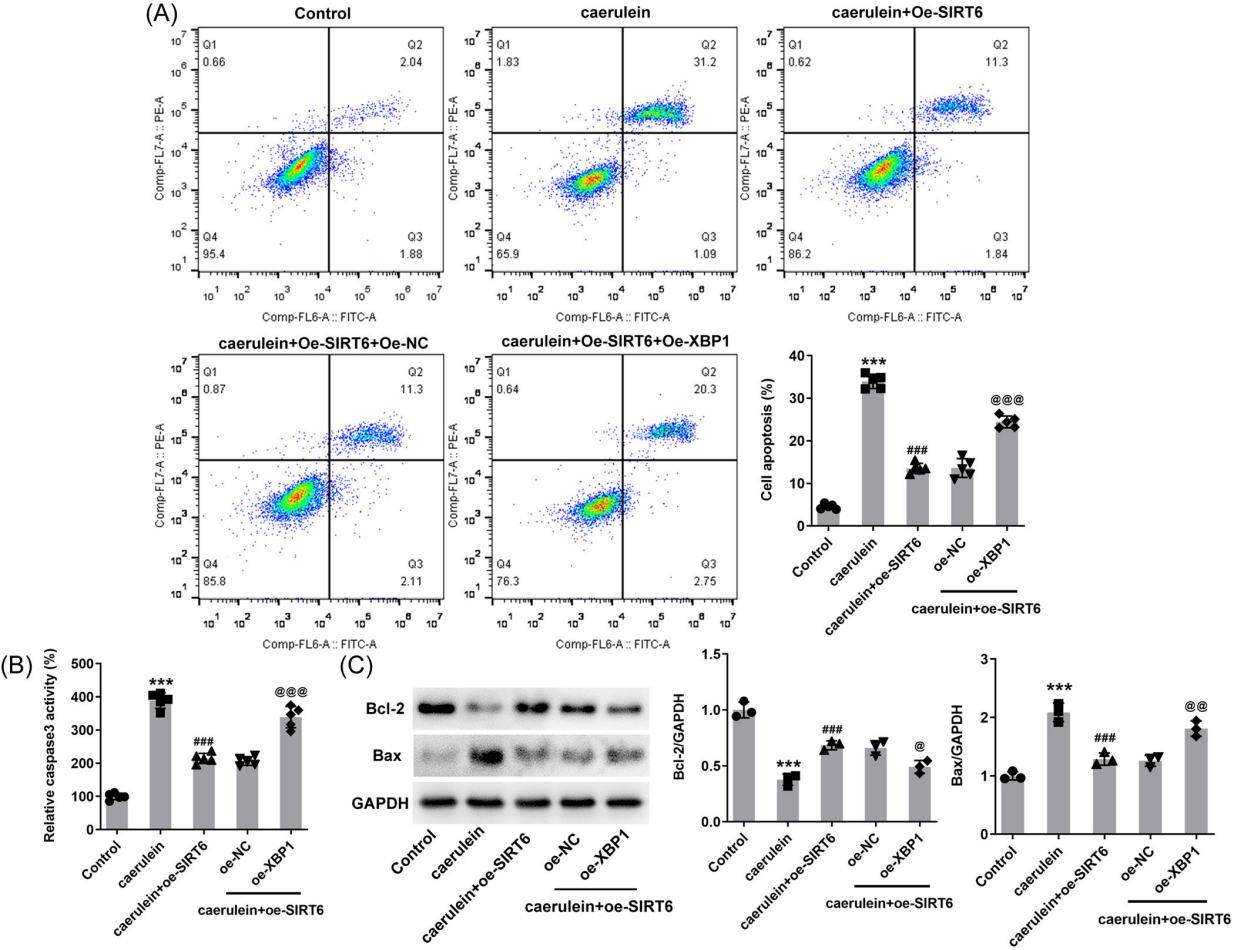
X‐Box binding protein 1 (XBP1) curbs the impacts of SIRT6 on the apoptosis. Cells were subjected to simultaneous transfection with SIRT6 and XBP1 overexpression plasmids. (A) Flow cytometry was used to detect cell apoptosis. (B) Caspase 3 activity was also used to assess apoptosis. (C) Western blot analysis reveals the protein levels of Bcl‐2 and Bax. ***p < .001 versus control; ^###^ *p* < .001 versus caerulein; ^@^ *p* < .05, p@@ < .01, p@@@ < .001 versus caerulein + oe‐SIRT6 + oe‐NC.

Continuing with the above grouping, additional transfection of XBP1 overexpression plasmid also caused an increase in the secretion levels of inflammatory factors (Figure 5A), accompanied by an increase in reactive oxygen species (ROS) (Figure 5B) and MDA contents and a decrease in SOD and CAT activities (Figure 5C). Upon caerulein treatment, SIRT6 overexpression reduced the apoptotic rate to ∼13%, while additional XBP1 overexpression increased the apoptotic rate to ∼23% (Figure 6A). In addition, caspase 3 activity (Figure 6B) and Bax protein levels increased due to XBP1 overexpression, and Bcl2 protein levels decreased (Figure 6C).

## 4 DISCUSSION

Pancreatitis stands as the most prevalent gastrointestinal disorder,^17^ with etiological factors encompassing gallstone‐induced pancreatic duct obstruction, acinar cell impairment induced by alcohol and specific drugs, pancreatic tissue trauma resulting from medical procedures or autoimmune conditions, as well as some idiopathic sources.^18^ The histological alterations observed in caerulein‐induced AP in rodents closely parallel those observed in human AP,^19^ rendering this modeling approach noninvasive, highly reproducible, and broadly applicable. In recent years, caerulein has gained widespread utilization in the establishment of AP animal models and is also well‐suited for cell‐based models. Our investigation revealed a reduction in SIRT6 levels in HPDE cells upon caerulein induction. Subsequently, to elucidate the specific role of SIRT6, intracellular SIRT6 overexpression was promoted through cell transfection. This augmentation of SIRT6 led to a notable reduction in the secretion of inflammatory factors, diminished apoptosis, and alleviated ER stress. Notably, the release of pro‐inflammatory cytokines plays a pivotal role in the pathogenesis of AP and can exacerbate local inflammatory responses.^20^ Subsequent SIRS and MODS are primary contributors to early mortality in AP. Thus, dampening the inflammatory response assumes paramount importance in disease management. Additionally, SOD and CAT were also observed to be activated upon SIRT6 overexpression, and the potential mechanism may be that SIRT6 catalyzes the deacetylation of SOD and CAT. As in the previous studies, the same family SIRT3 has been found to deacetylate lysine in manganese SOD or SOD2, resulting in increased enzyme activity.^21^, ^22^ Previously, in the context of diabetes, SIRT6 in pancreatic β‐cells has been shown to regulate insulin secretion in response to glucose stimulation, reducing cellular dysfunction and apoptosis.^14^ β‐Cell‐specific SIRT6‐deficient mice have reduced insulin secretion.^23^ These indicate that SIRT6 plays a key role in maintaining β‐cell function and vitality, and even in pancreatic health.

Recurrences of AP may progress into chronic pancreatitis, culminating in symptoms such as pain, indigestion, diabetes, and an elevated risk of pancreatic cancer.^24^, ^25^ Previous literature has expounded on the beneficial effects of upregulating SIRT6 in reducing glycolysis, epithelial‐mesenchymal transition, and distant metastasis in pancreatic cancer cells.^26^ Additionally, hypertriglyceridemia (HTG) ranks among the three common triggers of AP^27^ and HTG‐associated AP tends to be accompanied by severe pancreatic and organ failure.^28^ Previous findings indicated upregulation of UPR components, GRP78, CHOP, along with XBP‐1 in HTG, potentially exacerbating ER stress and promoting AP.^29^ Consequently, from a pathological standpoint, exploring strategies to mitigate ER stress for the treatment of AP, including increasing SIRT6 levels, appears feasible. Limited research suggests the regulatory mechanism between SIRT6 and ER stress. One study demonstrated that SIRT6 overexpression depleted histone H3 lysine 56 acetylation (H3K56ac) of the negative regulator of reactive oxygen species, thereby increasing ROS production. Subsequently, accumulated ROS activated ER stress.^30^ To the best of our knowledge, no other study has revealed the mechanism by which SIRT6 regulates ER stress, which requires more exploration in the future.

HPDE cells constitute the entirety of the pancreatic ducts and play a crucial role in secreting water, mucus, and electrolytes. These cells, along with the mucus they secrete, form the PDMB.^31^ Under normal physiological conditions, the PDMB serves as both a safeguard against the ingress of bile and trypsin into the pancreatic parenchyma and a pivotal checkpoint for regulating the secretion of zymogens by acinar cells.^28^ When stimulated, PDMB damage leads to widening of the intercellular spaces. Breach of PDMB integrity constitutes a pivotal early event in AP pathogenesis.^32^ Hence, preserving the normal physiological function of HPDE cells can mitigate early‐stage PDMB damage and contribute to AP treatment. This study revealed that XBP1 overexpression destroyed the protection of SIRT6 on HPDE cells, suggesting that upregulation of XBP1 could enable AP. Previous studies have demonstrated that XBP1‐deficient mice exhibited moderate hyperglycemia and glucose intolerance, with reduced insulin secretion originating from β‐cells.^33^ XBP1‐deficient mice were more susceptible to ethanol‐induced ER disorders and acinar cell pathology.^34^ These indicates that XBP1 contributes to the normal function of the pancreas and resistance to risk factors upon normal physiological conditions. Combined with XBP1 being upregulated in AP, it suggests that XBP1 homeostasis is essential for natural physiology.

In summary, our study illuminates the role of XBP1 in downregulating SIRT6 in HPDE cells, thereby promoting injury. Inhibiting XBP1 or augmenting SIRT6 levels holds promise in preserving cell function and represents a potential therapeutic avenue in the management of AP. However, the finding of the present study only supports the function of SIRT6 in regulating ER stress, and the precise signaling and the entire regulatory network between them are currently unclear. In addition, there are various types of cells in the pancreas, and the role and mechanism of XBP1 in other types have not been studied, including its verification in vivo.

## AUTHOR CONTRIBUTIONS

**Zhuo Yang**: Conceptualization; investigation; writing—original draft. **Shaojun Li**: Conceptualization; investigation. **Chuan Zhao**: Formal analysis; investigation. **Zongzheng Zhao**: Formal analysis; investigation. **Juan Tan**: Formal analysis; investigation. **Lu Zhang**: Formal analysis; investigation.

## CONFLICT OF INTEREST STATEMENT

The authors declare no conflict of interest.

## Data Availability

The data sets used and/or analyzed during the present study are available from the corresponding author upon reasonable request.
